# FLAIR-hyperintense lesions in anti-MOG-associated encephalitis with seizures presenting with postictal purpura and rhabdomyolysis: a case report

**DOI:** 10.3389/fimmu.2026.1827552

**Published:** 2026-08-05

**Authors:** Shang Xiang, Daiping Hua, Shi Yang, Youfa Huang, Lanting Sun, Bing Han, Wenming Yang, Han Wang

**Affiliations:** 1 Department of Neurology, The First Affiliated Hospital of Anhui University of Chinese Medicine, Hefei, China; 2 Department of Neurology, The Fifth Affiliated Hospital of Anhui University of Chinese Medicine, Lu’an, China; 3 Key Laboratory of Xin’an Medicine, Ministry of Education, Hefei, China

**Keywords:** case report, epilepsy, FLAIR-hyperintense lesions in anti-MOG-associated encephalitis with seizures, postictal purpura, rhabdomyolysis

## Abstract

FLAIR-hyperintense lesions in anti-MOG-associated encephalitis with seizures (FLAMES) represent a rare and distinctive clinico-radiological syndrome. Here, we report the case of a 35-year-old man who presented with sudden-onset generalized tonic-clonic seizures. Brain magnetic resonance imaging revealed cortical FLAIR hyperintensities in the right parietal lobe. During hospitalization, he developed scattered petechiae over the neck and chest, accompanied by elevated creatine kinase (CK) levels, findings that initially raised concern for systemic vasculitis or inflammatory myopathy. Given the close temporal relationship to the seizures, together with negative autoimmune and infectious evaluations, these abnormalities were ultimately interpreted as postictal purpura and seizure-associated rhabdomyolysis. Subsequent investigations confirmed serum positivity for anti-MOG antibodies, while antinuclear antibody profiling, vasculitis-related markers, and microbiological screening were all negative. After high-dose intravenous methylprednisolone therapy, seizure activity was controlled, the petechial rash resolved, CK levels normalized, and the cortical lesions showed marked regression on follow-up imaging. This case highlights that FLAMES may be complicated by postictal purpura and rhabdomyolysis, underscoring the potential for diagnostic confusion with systemic inflammatory disease.

## Introduction

Myelin oligodendrocyte glycoprotein antibody-associated disease (MOGAD) is now widely recognized as a distinct inflammatory demyelinating disorder of the central nervous system (CNS), separate from neuromyelitis optica spectrum disorder (NMOSD) and multiple sclerosis (MS) (1, 2). In 2017, a highly specific clinico-radiological phenotype of MOGAD was identified: FLAIR-hyperintense lesions in anti-MOG-associated encephalitis with seizures (FLAMES). This condition is characterized by cortical fluid-attenuated inversion recovery (FLAIR) hyperintensity, meningeal enhancement, and frequent seizures (3). While the neurological hallmarks of FLAMES are well documented, its secondary systemic manifestations are rarely described. In clinical settings, the concurrent presentation of encephalitic symptoms, cutaneous purpura, and elevated muscle enzymes typically prompts physicians to suspect multi-organ systemic conditions, such as systemic lupus erythematosus (SLE), antineutrophil cytoplasmic antibody (ANCA)-associated vasculitis, or severe sepsis. Such diagnostic misdirection can result in unnecessary invasive procedures and delay the initiation of targeted immunotherapy.

## Case presentations

A 35-year-old male was admitted to our neurology department two hours after experiencing a sudden, transient loss of consciousness accompanied by limb convulsions. The initial episode occurred at home and was characterized by sudden unresponsiveness, generalized limb twitching, upward eye deviation, jaw clenching, and tongue biting. There was no associated sphincter incontinence, nausea, or vomiting. The seizure lasted approximately two minutes before spontaneous resolution. Upon regaining consciousness, the patient complained of dizziness. He was subsequently brought to our outpatient clinic by his family and admitted with a provisional diagnosis of epilepsy. On admission, the patient was alert and oriented. His speech was coherent, and motor function in all four extremities was grossly intact. His baseline diet, sleep, and bowel/bladder functions were unremarkable. At approximately 05:00 the following morning, the patient experienced a second episode of loss of consciousness and limb convulsions. He was immediately placed on cardiac monitoring and received supplemental oxygen alongside intravenous diazepam (5 mg), which successfully aborted the attack; no further seizures were observed. During the ictal phase, his blood pressure was 170/100 mmHg, and his heart rate reached 120 beats/min (sinus rhythm). Both vital signs normalized post-ictally. Following the second seizure, scattered petechiae were noted on the patient’s neck and anterior chest (**Figure 1**). A dermatology consultation identified the lesions as purpura. Concurrently, the patient reported new-onset bilateral lower extremity weakness accompanied by myalgia in the medial thighs.

**Figure 1:**
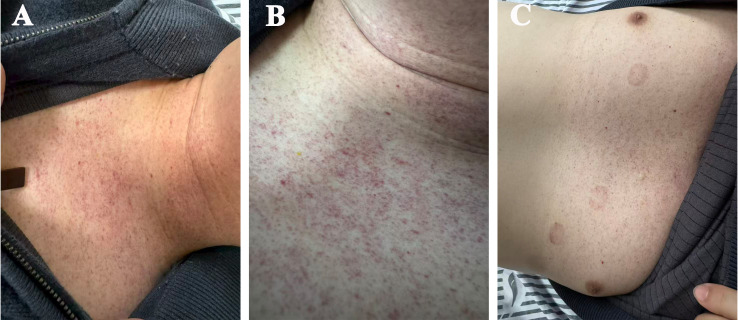
Purpura on the neck and chest. **(A,B)** Petechiae on the anterior and lateral regions of the neck. **(C)** Scattered petechiae on the anterior chest.

The patient’s past medical history was notable for a one-year history of hypertension (peak blood pressure: 160/110 mmHg), which was managed with a daily maintenance regimen of amlodipine/benazepril and carvedilol. He denied any other chronic conditions (such as diabetes mellitus or cardiovascular disease), infectious diseases (including hepatitis and tuberculosis), prior surgeries, major trauma, or blood transfusions. He had no known food or drug allergies, and his vaccination status was unverified. Regarding his social history, he was a lifelong resident with a 10-year smoking habit (approximately half a pack per day). He reported no alcohol consumption, illicit drug use, or exposure to parasitic infections or contaminated waters. He was married with healthy children, and his family history was unremarkable for any neurological, autoimmune, or genetic disorders.

Neurological examination upon admission revealed an alert, fully oriented, and cooperative patient. Cranial nerve evaluation was unremarkable; specifically, the pupils were equal, round (3 mm in diameter), and briskly reactive to light, with full extraocular movements, symmetrical facial expressions, an intact gag reflex, and a midline tongue protrusion. Motor examination demonstrated normal muscle tone and full strength in all extremities. Deep tendon reflexes were symmetrical. Sensory modalities and cerebellar coordination were grossly intact, and there were no signs of meningeal irritation.

On hospital day 2, extensive laboratory investigations—including a complete blood count, coagulation profile, C-reactive protein, hepatic and renal function tests, a vasculitis panel, immunoglobulins, antinuclear antibodies (ANA), and thyroid function, along with its autoantibodies—were all within normal limits. However, the patient’s creatine kinase (CK) level was markedly elevated at 1229.52 U/L (reference range: 46–171 U/L). Creatine kinase-MB (CK-MB) was 18.27 U/L (reference range: 0–24 U/L), myoglobin was elevated to 386 ng/mL (reference range: 28–72 ng/mL), and cardiac troponin T (cTnT) was < 0.01 ng/mL (reference range: < 0.014 ng/mL). Electroencephalography (EEG) revealed moderate-amplitude sharp-slow and spike-wave discharges originating in the right parietal region, with propagation to the right central region (supplementary material). Non-contrast brain magnetic resonance imaging (MRI) demonstrated a patchy lesion involving the right parietal cortex and subcortex. This lesion appeared slightly hypointense on T1-weighted imaging (T1WI) and hyperintense on both T2WI and FLAIR sequences. Diffusion-weighted imaging (DWI) showed no restricted diffusion, and the apparent diffusion coefficient (ADC) map displayed slightly elevated signals with ill-defined margins. To further characterize the lesion, contrast-enhanced MRI and susceptibility-weighted imaging (SWI) were performed. Post-contrast imaging revealed abnormal linear enhancement, while SWI demonstrated mild hypointensity in the corresponding cortical area, surrounded by multiple prominent, small dilated vessels (**Figure 2**). Given the clinical presentation and radiological features, an inflammatory etiology was highly suspected. A lumbar puncture was performed, revealing an opening pressure of 190 mmH_2_O. Routine cerebrospinal fluid (CSF) analysis showed clear and colorless fluid, with a white blood cell count of 2.00 × 10^6^/L and a red blood cell count of 0.00 × 10^6^/L. CSF biochemical analysis showed a glucose level of 4.78 mmol/L, a chloride level of 118.61 mmol/L, an adenosine deaminase level of 1.22 U/L, and a total protein concentration of 321.00 mg/L. To rule out immune-mediated and infectious causes, serum and CSF samples were sent for autoimmune encephalitis (AE) panels, CNS demyelinating disease antibodies, and next-generation sequencing (NGS). In the interim, the patient was initiated on oral levetiracetam (500 mg, twice daily) for seizure control, alongside potassium supplementation and close clinical monitoring of CK levels and cutaneous purpura.

**Figure 2:**
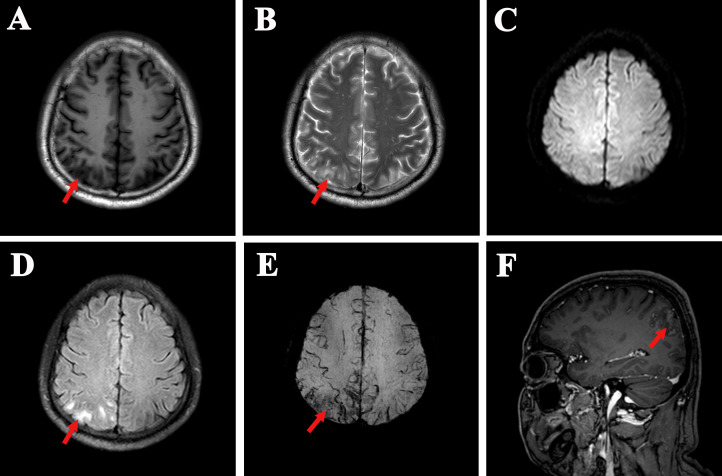
**(A-F)** The first head MRI results. The red arrow indicates abnormal intracranial signals.

On hospital day 3, targeted NGS of the CSF returned negative for any specific pathogens, including bacteria, fungi, viruses, and parasites. The AE panels for both serum and CSF were also negative. However, the serum CNS demyelinating panel revealed the presence of MOG antibodies at a titer of 1:100.

According to the 2023 international diagnostic criteria for MOGAD (4), the diagnosis requires (1): a typical demyelinating clinical attack involving the CNS (2); positive serum MOG immunoglobulin G (IgG) antibodies detected by cell-based assay (CBA); and (3) exclusion of alternative diagnoses. Our patient fulfilled all three criteria. First, the clinical presentation was consistent with a typical CNS demyelinating event, manifesting as encephalitis with epileptic seizures. Second, serum MOG IgG antibodies were positive by CBA, with a titer of 1:100. Third, systematic investigations excluded other suspected diseases. Specifically, metagenomic NGS of CSF, autoantibody panels for AE in both serum and CSF, and microbiological studies were all negative. These results effectively ruled out MS, NMOSD, CNS infections, autoimmune encephalitis, and systemic autoimmune diseases. Taken together, the final diagnosis was MOGAD presenting as FLAMES. Following the diagnosis, the therapeutic regimen was adjusted to intravenous methylprednisolone pulse therapy, administered at 1, 000 mg/day for 3 days, followed by a tapering schedule of 500 mg/day for 3 days, 240 mg/day for 3 days, and 120 mg/day for 3 days. The patient was subsequently transitioned to oral prednisone at 60 mg/day, with a planned taper of 5 mg every 2 weeks, aiming for a 6-month maintenance period. Before discharge, the patient remained completely seizure-free, the cutaneous purpura resolved, and serum CK levels normalized. A follow-up brain MRI demonstrated a significant reduction in the size of the right parietal lesion (**Figure 3**). The clinical timeline is detailed in **Figure 4**.

**Figure 3:**
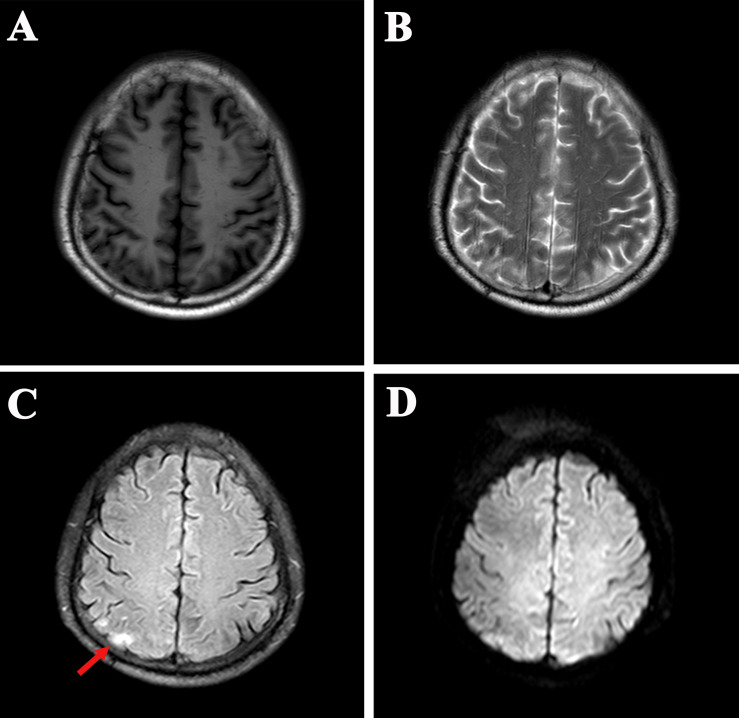
**(A-D)** Pre-discharge follow-up head MRI results. The red arrow indicates abnormal intracranial signals.

**Figure 4:**
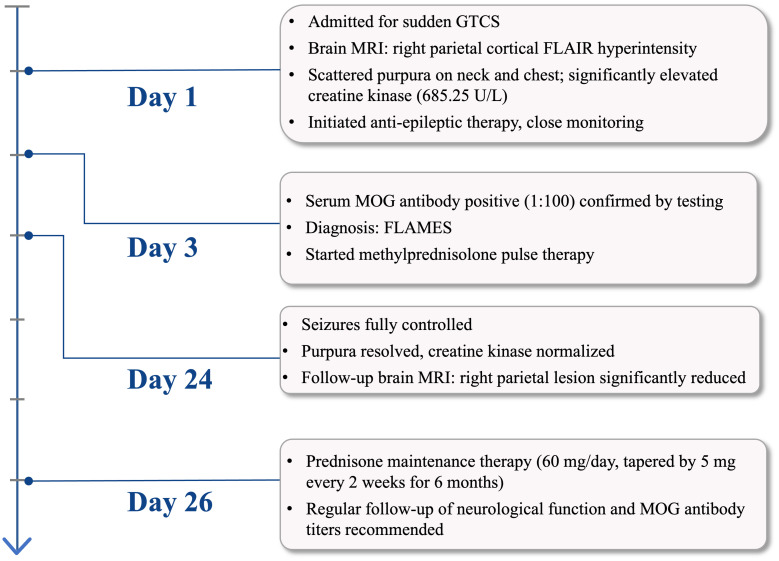
Patient clinical course timeline.

## Discussion

MOGAD encompasses a spectrum of inflammatory demyelinating disorders of the CNS characterized by diverse clinical phenotypes (2, 5). Epidemiological data regarding the incidence and prevalence of MOGAD remain limited. Available European studies have estimated its incidence to be approximately 1.6 ~ 3.4 cases per million person-years (6). Unilateral cortical FLAMES has recently been recognized as a rare yet highly specific clinico-radiological syndrome within the MOGAD spectrum (3, 7). Our patient presented with a sudden-onset GTCS. Brain MRI revealed T2/FLAIR hyperintensities in the right parietal cortex and subcortical regions without restricted diffusion on DWI, alongside linear leptomeningeal enhancement on contrast sequences. These findings perfectly align with the classic neuroimaging hallmarks of FLAMES. Furthermore, SWI demonstrated multiple engorged, fine vessel signals surrounding the lesions. This phenomenon likely reflects localized cortical venous congestion and blood-brain barrier disruption, serving as a crucial imaging marker to support an inflammatory demyelinating etiology and differentiate it from acute ischemic stroke. Notably, the patient’s routine CSF profile, biochemical markers, and autoimmune antibody panels were entirely unremarkable; the definitive diagnosis ultimately relied on serum MOG-IgG positivity. This observation corroborates previous reports suggesting that MOG antibodies are primarily produced peripherally, making serum testing significantly more sensitive than CSF assays (8–10). Consequently, in cases with a high clinical suspicion of FLAMES, serum MOG antibody testing should be prioritized to avoid missed diagnoses and therapeutic delays. Following definitive diagnosis, the patient achieved seizure freedom and radiological regression of the lesions upon receiving high-dose intravenous corticosteroid pulse therapy, further underscoring the underlying immune-mediated inflammatory nature of the disease.

Upon initial admission, the patient presented with bilateral lower-extremity weakness and myalgia, accompanied by markedly elevated serum CK levels, indicating rhabdomyolysis. Given the inflammatory nature of MOGAD, it is important to distinguish whether rhabdomyolysis represents direct inflammatory muscle involvement or a secondary consequence of epileptic seizures. Although rhabdomyolysis has been reported in AE, particularly in association with severe agitation, dyskinesia, autonomic instability, or prolonged seizures (11, 12), there is currently no evidence suggesting that rhabdomyolysis is a direct clinical manifestation of MOGAD itself. In addition, the patient showed no persistent proximal muscle weakness, skin manifestations suggestive of dermatomyositis, sustained elevation of muscle enzymes independent of seizures, or other clinical features supporting primary inflammatory myopathy or mitochondrial encephalomyopathy.

In the present case, several findings support seizure-induced rhabdomyolysis. First, the elevation of CK occurred in close temporal association with GTCS. Second, the muscle pain predominantly appeared after convulsive episodes, consistent with postictal muscle injury. Third, after antiseizure treatment and corticosteroid therapy controlled the cortical inflammatory lesions and prevented further seizures, the patient’s myalgia improved rapidly, and CK levels gradually normalized. Mechanistically, generalized tonic-clonic seizures can cause intense, sustained, and involuntary skeletal muscle contractions, leading to excessive adenosine triphosphate consumption, sarcolemmal injury, and subsequent leakage of intracellular contents, including CK and myoglobin, into the circulation (13).

Accordingly, secondary rhabdomyolysis should be recognized as a potential accompanying complication during the acute phase of FLAMES when major motor seizures occur. Clinicians should monitor serum CK, myoglobin, electrolytes, and renal function in patients presenting with prolonged or recurrent generalized seizures, and promptly initiate hydration and supportive management to prevent complications such as electrolyte disturbances and acute kidney injury (14).

The most diagnostically challenging feature of this case was the presence of scattered purpura over the neck and anterior chest. The simultaneous occurrence of encephalitis and purpuric skin lesions prompts a broad differential, encompassing ANCA-associated vasculitis, SLE, antiphospholipid syndrome, immune-complex–mediated small-vessel vasculitis, and fulminant hematogenous CNS infections such as meningococcal disease (15). Drug-induced purpura, thrombocytopenic purpura, and acquired coagulopathies warrant equal consideration.

In the present case, platelet counts and coagulation profiles were unremarkable. Vasculitis autoantibody panels and metagenomic NGS for infectious pathogens were uniformly negative. Critically absent were systemic features of vasculitis–renal impairment, pulmonary hemorrhage, sustained fever, arthritis, or serological evidence of autoimmune disease. While immune-mediated endothelial injury cannot be entirely excluded on theoretical grounds, the salient observation was the spontaneous, complete resolution of the cutaneous lesions following seizure cessation, without any targeted dermatological intervention.

To our knowledge, no prior report has described purpura as a concurrent manifestation of MOGAD. Taken together, the characteristic thoracocervicofacial distribution, entirely normal hematological and coagulation parameters, self-limited clinical course, and tight temporal coupling with convulsive seizure activity point to a diagnosis of postictal purpura (16–18). The underlying mechanism reflects the acute hemodynamic consequences of tonic–clonic seizures: sudden, marked elevation of intrathoracic and central venous pressure–driven by Valsalva-like straining, transient apnea, and paroxysmal coughing–precipitates rupture of superficial dermal capillaries and extravasation of erythrocytes into the dermis. Recognizing this phenomenon carries direct clinical importance: it enables the clinician to reframe an apparently multisystemic presentation, avoid unnecessary invasive workup such as skin biopsy or bone marrow examination, and redirect diagnostic and therapeutic efforts toward the underlying CNS pathology and MOGAD-specific immunotherapy.

## Conclusion

This study reports a rare case of FLAMES syndrome. The patient initially presented with GTCS, subsequently complicated by rhabdomyolysis and postictal purpura. This case highlights that when patients exhibit concurrent CNS involvement, purpura, myalgia, and significantly elevated CK levels, clinicians should maintain a high index of suspicion for secondary muscle injury and metabolic disturbances caused by severe epileptic seizures, in addition to routinely screening for multisystem autoimmune vasculitis or severe systemic infections. Correctly recognizing these GTCS-derived secondary signs enables physicians to look past complex systemic manifestations and identify the underlying CNS pathology. Consequently, this helps avoid unnecessary invasive procedures or excessive testing, ensuring the timely administration of targeted immunomodulatory and antiseizure therapies. Despite providing valuable insights into clinical differential diagnosis, this study has certain limitations. First, as a single case report, it lacks validation from multicenter, large-cohort data. Second, because the purpura and rhabdomyolysis resolved rapidly and spontaneously once the seizures were controlled, skin and muscle biopsies were omitted to minimize invasiveness and avoid over-medicalization. Finally, this report focuses primarily on acute-phase diagnosis and management, lacking long-term follow-up data for the maintenance and post-treatment periods. The patient’s long-term neurological recovery, the dynamic changes in MOG antibody titers, and the potential risk of disease relapse warrant continuous monitoring and evaluation in future clinical practice.

## Patient perspective

The patient stated: “The sudden seizures, purple spots, and severe muscle pain were deeply distressing. I initially feared a life-threatening systemic disease. However, identifying the MOG antibody provided clarity and relief. I am grateful for the precise diagnosis that avoided unnecessary invasive tests. I hope my experience helps clinicians recognize these symptoms to ensure other patients receive timely and effective care.”

## Data Availability

The original contributions presented in the study are included in the article/supplementary material. Further inquiries can be directed to the corresponding author.
